# Pharmaceutical Cocrystal Formation of Pyrazinamide with 3-Hydroxybenzoic Acid: A Terahertz and Raman Vibrational Spectroscopies Study

**DOI:** 10.3390/molecules24030488

**Published:** 2019-01-30

**Authors:** Qiqi Wang, Jiadan Xue, Zhi Hong, Yong Du

**Affiliations:** 1 Centre for THz Research, China Jiliang University, Hangzhou 310018, China; erwangqiqier@126.com (Q.W.); hongzhi@cjlu.edu.cn (Z.H.); 2 Department of Chemistry, Zhejiang Sci-Tech University, Hangzhou 310018, China; jenniexue@126.com

**Keywords:** pyrazinamide, 3-hydroxybenzoic acid, terahertz time-domain spectroscopy, Raman spectroscopy, density functional theory

## Abstract

Vibrational modes of pyrazinamide (PZA), 3-hydroxybenzoic acid (3-hBA), and their cocrystal were characterized using terahertz time-domain (THz-TDS) and Raman vibrational spectroscopic techniques. In experimental THz spectra, the cocrystal has characteristic absorption bands at around 0.81, 1.47, and 1.61 THz, respectively, meanwhile the raw materials are absolutely different in this region. Raman spectra also show similar results about differences between the cocrystal and corresponding starting parent materials. Density functional theory (DFT) was used to simulate both optimized structures and vibrational modes of the cocrystal formed between PZA and 3-hBA. The vibrational modes of such cocrystal are assigned through comparing the simulation DFT frequency results with experimental vibrational spectra. The calculation of the theoretical THz spectrum shows that the hydrogen bonding effect established between H11–N12–H13 and the carboxyl group -COOH makes contributions to the formation of absorption peaks in 0.49, 0.62, 0.83, and 1.61 THz, which agrees pretty well with experimental results. The theoretical Raman result also matches well with experimental observations. The results provide a fundamental benchmark for the study of pharmaceutical cocrystal formation and also inter-molecular hydrogen bonding interactions between active pharmaceutical ingredients and various cocrystal coformers based on Raman and terahertz vibrational spectroscopic techniques combined with theoretical simulations.

## 1. Introduction

A lot of active pharmaceutical ingredients (APIs) have been eliminated from late-stage development as drug candidates because of their poor physicochemical properties, especially solubility, which poses a serious threat to clinical applications [1]. Compared to their parent APIs, pharmaceutical cocrystals formed from the combination between APIs and various cocrystal conformers (CCFs, being included on a Generally Recognized as Safe list or pharmaceutically acceptable coformers) show unique physicochemical properties with improved dissolution rate, solubility, hygroscopicity, mechanical property, and also photostability [2,3,4,5,6]. Cocrystallization has special potential in improving characteristics and therapeutic utilities of drugs without altering the chemical nature and bioactivity of specific APIs [7]. Such features make research about cocrystal formation and corresponding improvement of physicochemical properties to be considered as a promising alternative to several dissolution-assisting technologies involving amorphous solids, salt crystals, solvates, and polymorphs, which provide adequate bioavailability to oral formulations comprising low-solubility APIs. Therefore, characterization and detection of such cocrystal formations plays an essential role in the field of pharmaceutical research and development.

**Figure 1:**
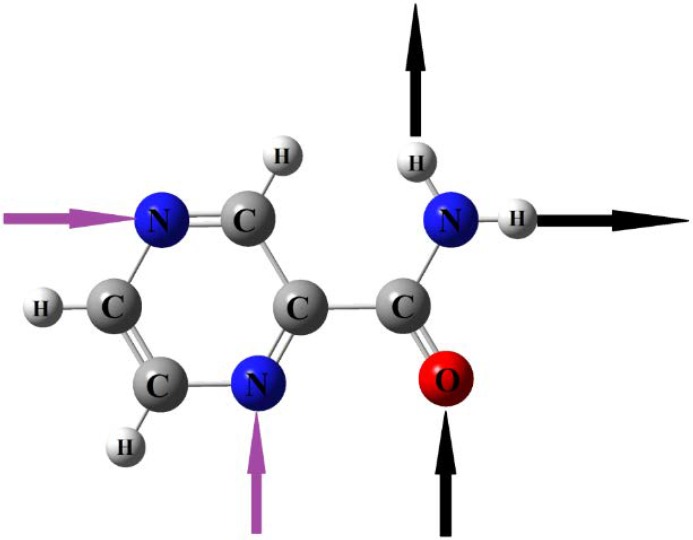
Molecular structure of pyrazinamide (PZA) (black arrows represent hydrogen bond donor positions and purple arrows indicate the possible positions of the hydrogen bond acceptor).

Pyrazinamide (PZA) is an important front-line medicine for treatment of tuberculosis (TB). As a typical anti-tubercular drug, PZA could be used in monotherapy as well as fixed dose combination (FDC) along with rifampicin and ethambutol, affording the most effective tuberculosis chemotherapy clinically recommended by the World Health Organization [8]. PZA owns multiple hydrogen-bonding sites, including a primary amide group and N-containing aromatic hetero-molecular (with the molecular structure shown in Figure 1, in which black arrows represent hydrogen bond donor positions and purple arrows indicate the possible positions of the hydrogen bond acceptor). PZA consists of a five-member N–H…N intramolecular hydrogen bond between the amide N–H and the adjacent N atom of the pyrazine ring. In tuberculosis diseases, tissue inflammation and free radical bursts from macrophages results in oxidative stress. Without anti-oxidants, free radicals would cause pulmonary inflammation [9,10,11]. The ability to resist the oxidation of APIs plays a critical role in the shelf-life of a pharmaceutical formulation. Hydroxybenzoic acids are typical well-known anti-oxidants, which can prevent the oxidation process [12]. Zaworotko et al. [13] reported that the primary amide functional group of PZA and 2,5-dihydroxybenzolic acid formed supramolecular hetero-synthon through the specific inter-molecular hydrogen bonding effect, and they found that corresponding pharmaceutical co-crystals could offer an opportunity to increase the number of forms of an API and also address its poor solubility. Abourahma et al. [14] employed differential scanning calorimetry (DSC), powder X-ray diffraction (PXRD), single crystal X-ray diffraction (SC-XRD), and nuclear magnetic resonance (NMR) to study cocrystal formation of PZA and hydroxybenzoic acid. Their results indicated that indeed all hydroxybenzoic acids are pretty suitable CCFs for PZA. Lou et al. [15] performed Hirshfeld surfaces and PXRD to analyze series cocrystal structures of PZA with 3-hydroxybenzoic acid (3-hBA), 4-hydroxybenzoic acid (4-hBA), and 3,4-dihydroxybenzolic acid (3,4-DHBA). Based on their experimental and theoretical analysis, they deduced that the strong O–H…O, N–H…O inter-molecular hydrogen bonding interactions between PZA and hydroxy-substituted benzoic acids made the structures of co-crystals stabilized. Although cocrystallizing with the two drugs, namely PZA and isoniazid, into a single crystal directly has so far not been successful, in Cherukuvada’s work [16] two 1:1:1 ternary cocrystals were reported between PZA and isoniazid along with the bridge help of two typical dicarboxylic acids, namely succinic acid and fumaric acid. The results indicated that such ternary cocrystals of PZA drugs exhibited optimized formulation capacity and also in vitro/vivo synergistic effects.

Various analytical techniques with specific advantages and disadvantages are used for solid-state material characterization [17]. However, investigations on the molecular structure of anti-tubercular of PZA with its various CCFs are rarely reported. Vibrational spectroscopy such as Raman and THz spectroscopy could provide rich information to understand the hydrogen bond effects on the molecular structures of specific materials. Raman spectroscopy is based on the inelastic scattering of photons by molecules and this analytic technique has been widely applied in analyzing, and also identifying, intra-molecular vibration and/or rotation information of molecular structures of various compounds [18]. Meanwhile, the emerging terahertz time-domain spectroscopy (THz-TDS) is pretty sensitive to the weak inter-molecular interactions, skeleton-vibration for materials, and also dipole transitions within low-frequency vibrational regions. What is more, THz spectroscopy has become a potential method to analyze pharmaceutical cocrystals both non-invasively and non-destructively as well as investigate the collective vibrations and the whole structural interactions shown within various materials [19,20]. Combining Raman with THz spectroscopy together, Du et al. [20] investigated molecular structures of the cocrystal formed between 5-fluorocytosine and fumaric acid, and also analyzed the hydrogen bonding effects during the forming of such a solid-state pharmaceutical cocrystallization process.

In this work, vibrational spectroscopy including THz absorption and Raman vibration spectra was used for the characterization of PZA, 3-hBA, physical mixture, and also their cocrystals. A quantum chemical density functional theory (DFT) calculation was used to optimize structures and simulate the vibrational frequencies of the cocrystal in order to help understand the Raman and THz spectroscopic observation and also structural changes, due to the inter-molecular interactions between PZA and 3-hBA by comparing with the experimental spectra results [21,22]. Combined vibrational spectroscopic results with DFT simulation calculations, rich information about molecular structures and inter-molecular hydrogen bonding interactions of the cocrystal formed between anti-tubercular drug PZA and 3-hBA, could be obtained successfully.

## 2. Results and Discussion

### 2.1. THz Absorption Spectral Characterization and Analysis of PZA, 3-hBA, Physical Mixture, and Their Cocrystals

**Figure 2:**
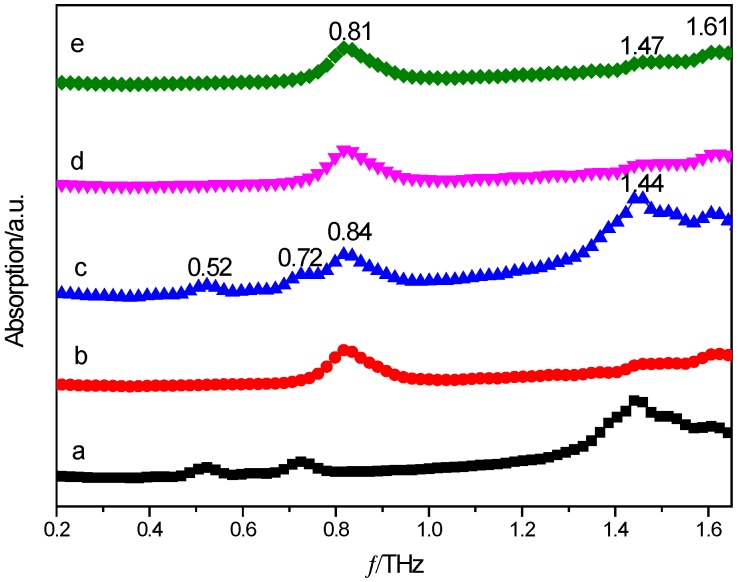
THz spectra of PZA (**a**); 3-hydroxybenzoic acid (3-hBA) (**b**); physical mixture (**c**); solvent cocrystal (**d**); and grinding cocrystal (**e**).

Figure 2 shows THz spectra of PZA, 3-hBA, physical mixture, and corresponding co-crystals prepared from solvent slow evaporation and neat grinding methods. It could be seen that the physical mixture has three characteristic peaks at 0.52, 0.72, and 1.42 THz, meanwhile, both of the cocrystals have two characteristic peaks at 0.81 and 1.47 THz. The characteristic peaks at 0.52 and 0.72 THz shown in the spectra of the physical mixture have disappeared in the spectra of cocrystal. At the same time, the physical mixture has a strong peak at 1.42 THz and has moved to 1.47 THz in that of both cocrystals. It could be clearly seen that the absorption peaks of cocrystals are distinctively different from those of the physical mixture, namely the simple linear superposition of the starting materials PZA and 3-hBA. THz absorption spectra of cocrystals between PZA and 3-hBA prepared from solvent slow evaporation (solvent cocrystal) and the neat grinding method (grinding cocrystal) are shown in Figure 2d,e. It could also be seen from the figure that the absorption peaks of solvent and grinding cocrystals appear at almost the same positions with obvious characteristic peaks at 0.81 and 1.47 THz, which indicates that these two cocrystals prepared with different methods should have almost the same molecular structures, and no impurities were formed under different experimental conditions. These spectral results indicate that THz spectroscopic technology could provide obvious fingerprint information for various solid-state crystalline molecular structures. The apparent differences can be explained reasonably by some theoretical calculations, which are effective methods for bridging observed vibrational modes and corresponding molecular structures shown in different crystalline materials [23].

**Figure 3:**
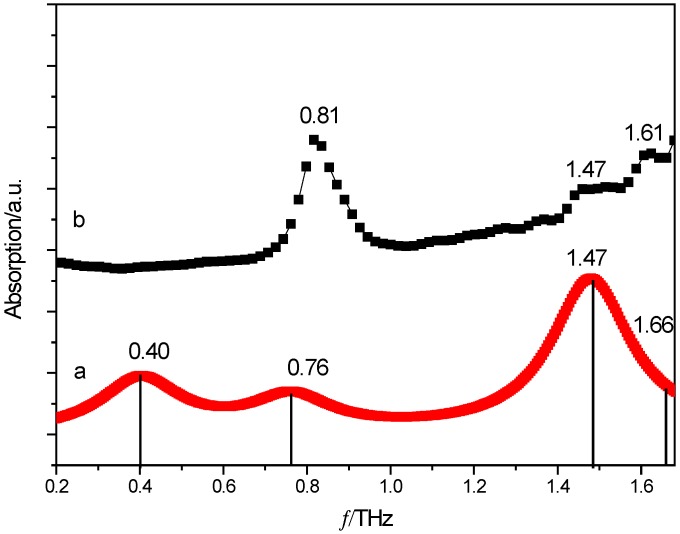
Comparison of THz spectra between simulated (**a**) and experimental (**b**) results of PZA and 3-hBA.

Comparison of the DFT theoretical simulation and experimental THz spectra of cocrystal between PZA and 3-hBA is shown in Figure 3. In the THz spectra, the molecular vibrational modes are mainly caused by deformation vibrations, bending vibrations, and twisting vibrations of those that participate by multiple functional groups being the components of specific materials. The positions of the peaks are different due to the various resonance responses of such vibrational modes presented in APIs and their corresponding cocrystals. In the diagram, there are four absorption peaks at the positions of 0.40, 0.76, 1.47, and 1.66 THz in the theoretical spectrum. It could be seen that the characteristic peaks at 0.81 and 1.47 THz in the experimental spectral result are in pretty good agreement with those at 0.80 and 1.53 THz in the simulated result. However, the absorption peaks of 0.40 THz in the theoretical spectra did not appear in the experimental spectrum, which may be the reasons that our calculation was based on the single-molecule structure and ignored inter-molecular forces within solid-state crystalline unit cells. Additionally, another possible reason is that the theoretical results were obtained under the condition of absolute 0 K while the experimental results are obtained under the room temperature condition (at around 298 K) [24,25].

**Figure 4:**
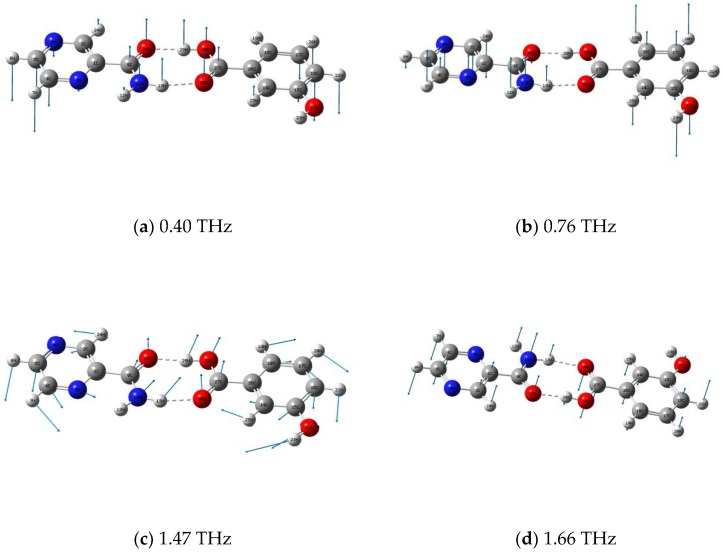
Vibrational modes description of cocrystal between PZA and 3-hBA at 0.40 (**a**); 0.76 (**b**); 1.47 (**c**); and 1.66 (**d**) THz.

Different vibrations make contributions to these peaks appearing at different positions. Through the dynamic observation help of Gaussian-view software, the vibrational modes description of assignments of the cocrystal between PZA and 3-hBA is shown in Figure 4, and the entire corresponding assignment could also be obtained, as shown in Table 1. The characteristic peak of the cocrystal between PZA and 3-hBA at 0.40 THz in the theoretical results is mainly due to 3-hBA and PZA molecular out of plane bending vibrations, as shown in Figure 4a. The experimental spectral feature at 0.76 THz arises from out of plane bending vibrations of R_1_ (the six ring in 3-hBA), R_2_ (the six ring in PZA), and H12–N11–H13 out of plane bending vibrations, as shown in Figure 4b. The experimental characteristic peak at 1.47 THz can be attributed to the calculated vibrational mode at 1.47 THz, arising from out of plane bending vibrations of PZA and 3-hBA molecular, as shown in Figure 4c. In addition, the characteristic peak of the cocrystal at 1.61 THz in experimental results is mainly due to 3-hBA and PZA molecular out of plane bending vibrations, as shown in Figure 4d.

According to the experimental and calculated results, the formation of hydrogen bonds causes some changes in the structure of PZA and 3-hBA involved in their cocrystals. Therefore, the THz absorption spectrum of cocrystal displays completely different characteristic peaks in the THz spectral region from those of the starting raw materials. The above combined analysis of experimental results and theoretical simulation show that the hydrogen bonding effect plays an important role in changing the molecular structure between PZA and 3-hBA upon its cocrystallization, and also indicates that these four characteristic absorption peaks shown in cocrystals arise from individual vibrational modes of such cocrystals due to strong inter-molecular interactions between PZA and 3-hBA.

### 2.2. Raman Spectral Characterization and Analysis of PZA, 3-hBA, Physical Mixture, and Their Cocrystals

**Figure 5:**
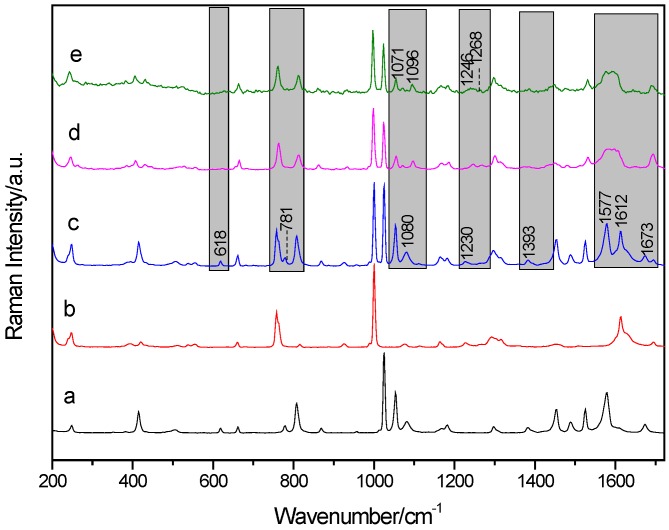
Raman spectra of PZA (**a**); 3-hBA (**b**); physical mixture (**c**); their solvent cocrystal (**d**); and grinding cocrystal (**e**).

Raman spectroscopy has become a crucial and mainstream measurement technique to provide specific structural fingerprint information by which different materials could be exclusively characterized at the micro-molecular level and also identified in various chemical processes [26]. Experimental Raman spectra of the two cocrystals between PZA and 3-hBA are shown in Figure 5d,e, and it can be easily seen that both solvent cocrystal and grinding cocrystal are almost the same as each other. It is confirmed that the crystalline phase of the cocrystal formed under the grinding condition is same as that of solvent cocrystal, which is also in agreement with the above reported THz spectral results. In contrast, Raman spectra of cocrystal formed between PZA and 3-hBA could be easily differentiated from that of the physical mixture and also the individual starting reactants, which can be seen in Figure 5. Several band shifts and new characteristic features were observed in the whole spectral 200~1800 cm^−1^ region. In the Raman spectrum of physical mixture, the characteristic peak at 618, 781, and 1383 cm^−1^ are almost disappearing in that of cocrystals. Regarding the Raman spectra of the cocrystals, the new characteristic bands have appeared at 1096 and 1268 cm^−1^, while the spectrum of the physical mixture or starting materials does not show any of them. The peak in the physical mixture at 1080 cm^−1^ has been red shifted to be at 1071 cm^−1^, while peaks at 1230 and 1297 cm^−1^ have been blue shifted to 1246 and 1300 cm^−1^, respectively, along with the formation of the cocrystal between PZA and 3-hBA. It could be primarily induced that the inter-molecular interaction, such as the strong hydrogen bonding effect, leads to the formation of these two cocrystals and changes the involved molecular structures of raw materials [3]. The obvious difference is the two strong peaks at 1577 cm^−1^ due to the contribution from PZA, and at 1612 cm^−1^ due to the contribution from 3-hBA in the spectrum of the physical mixture. However, these two strong peaks gradually move in the opposite direction and are finally assumed to be pretty broad peaks in the spectrum of cocrystal, which can be seen in Figure 5d,e. This evidence explains that the cocrystal between PZA and 3-hBA has been formed and the molecular conformation/configuration shown in the cocrystal is different from the raw materials.

**Figure 6:**
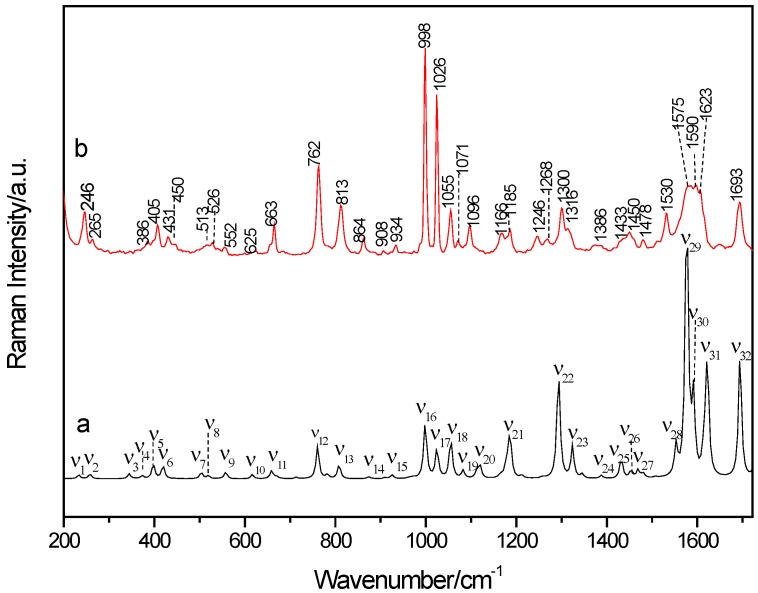
Comparison of Raman spectra between simulated (**a**) and experimental (**b**) results of the cocrystal between PZA and 3-hBA.

Comparison of the theoretical DFT calculation and the experimental Raman spectral results is shown in Figure 6 and it can be seen that the theoretical simulated spectrum is in pretty good agreement with the experimental result. The characteristic vibrational bands of the cocrystal shown in Raman spectra are listed in detail in Table 2 with complete band assignments. The characteristic peak at 1433 cm^−1^ in the experimental result is caused by scissor vibrations of H29–O28–C27=O30 and in plane vibrations of C5–H8, belonging to 3-hBA, and scissor vibrations from H13–N11–C9, which belongs to PZA. Scissor vibrations from H30=C27–O28–H29, stretching vibrations of C26–H27, and in plane vibrations of C16–H21 and C17–H20, belong to 3-hBA, and in plane vibrations of N11–H13, C4–H7, and C5–H8, belonging to PZA, make contributions to forming the characteristic peak at 1478 cm^−1^. At 1693 cm^−1^, this characteristic peak is originated from stretching vibrations from C9=H10 and scissor vibrations of H12–N11–H13, belonging to PZA, and scissor vibrations from C27–O28–H29 and stretching vibrations of C27=O30, from 3-hBA. The vibrational modes assignment of the above four characteristic peaks suggest that the hydrogen bonding formation in C27=O30…H13–N11 and C9=O10…H29–O28 between PZA and 3-hBA plays a main role in its cocrystal formation.

**Figure 7:**
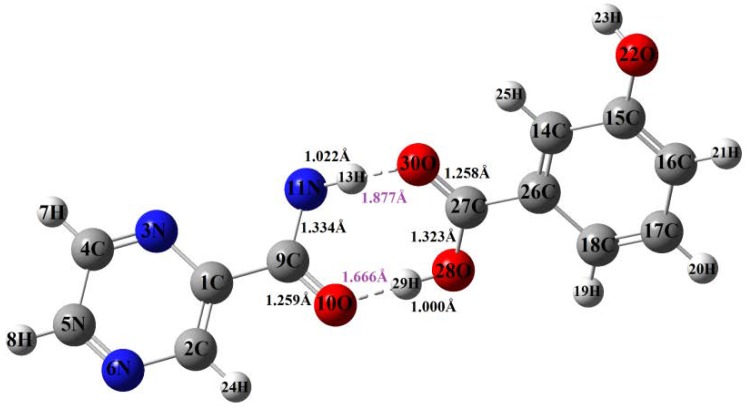
Typical bond lengths of the cocrystal between PZA and 3-hBA (bond length shown with unit Å).

The change of bond length is one of the most important factors which can help to understand the molecular structure changes upon pharmaceutical cocrystallization occurring. Figure 7 shows typical bond length changes of the cocrystal based on their DFT optimized structures. In addition, Table 3 shows the bond length change of intramolecular typical bonds and intermolecular hydrogen bonds in the PZA-3-hBA cocrystal form. Several bond lengths of PZA have been changed in cocrystal, and the formation of hydrogen bond length mainly occurred at the amino group in N11–H13 and the carbonyl group of C9=O10. Here, 3-hBA has a carboxylic group and the structure has changed in the cocrystal with the bond length of C–OH being shortened to be 1.431 Å from 1.323 Å, while the bond length of C=O and O–H has been elongated to be 1.258 and 1.000 Å from 1.225 and 0.963 Å, respectively. The bond length of N11–H13 has been extended from 0.998 Å to 1.022 Å, which has caused the Raman peaks’ movement and other changes. The C9–H10, C27=O30, O28–H29, and N11–H13 make important contributions to the vibrations of peaks in the positions of 1433, 1478, and 1386 cm^−1^, which are mainly attributed by the formation of hydrogen bonds through C7=O8 and N12–H11. Connecting the vibrational modes assignment and the changing of bond length, it could be more convenient to understand the reasons of the peaks’ appearance and disappearance shown in Raman experimental spectra.

According to the analysis of Raman and THz spectra, it shows that the formation of hydrogen bonds change the intra/inter-structure of molecules. Additionally, intra/inter-molecular vibration of PZA-3-hBA cocrystal could be achieved in detail by using the above two spectroscopic techniques. Compared with starting materials and the corresponding cocrystal, THz absorption spectra could more clearly reflect molecular configuration, which has been applied in security checks, pharmaceuticals, chemicals, biology, and so on.

## 3. Materials and Methods

### 3.1. Chemicals and Sample Preparation

The 3-hBA and PZA were purchased from Sigma-Aldrich (Shanghai, China) and used as received without further purification. All other chemicals were obtained from various commercial suppliers and used as received. The API (PZA) and CCF (3-hBA) samples were ground before mixing to achieve particles with the mean size of several micrometers in order to minimize the scattering effects from sample particles during THz spectral measurements.

The physical mixture was obtained by gently mixing two compounds with a 1:1 molar ratio in a vial using a vortex mixer for 10 min.

Grinding cocrystal was performed by co-grinding PZA with 3-hBA at 1:1 molar ratio in 25 mL stainless steel milling jars using a planetary ball mill (QM-3SP, gear type, Nanjing University Instrument Plant) with a frequency of 25 Hz at room temperature.

Solvent cocrystal was prepared by the slow solvent evaporation method. Equimolar PZA (0.123 g, 1 mmol) and 3-hBA (0.138 g, 1 mmol) were dissolved in an amount of methanol (20 mL). The solution was slowly evaporated at room temperature and the white snow-flake solvent cocrystal was obtained after several days.

All the samples were weighted into ~200 mg aliquots and poured into a steel die and subjected to ~4 MPa pressure for several minutes. The sample discs, ~13 mm in diameter and ~1.5 mm thick, were obtained and sealed in plastic bags before vibrational spectroscopic measurements.

### 3.2. Apparatus and Procedure

Raman spectra were obtained using a Fourier transform Raman spectrometer (Thermo Nicolet Corporation, Madison, WI, USA) with diode pumped solid-state laser (wavelength 1064 nm) as the near-infrared source. Spectra were acquired over 500 scans at 2 cm^−1^ resolution over the wavenumber range 150–3500 cm^−1^ with a laser operating power 150 mW. Total analysis time per sample was of the order of 6 min intervals.

A Z2 measurement system (Zomega Co. Ltd., New York, NY, USA) was adopted in THz-TDS. This standard THz-TDS setup based on photoconductive switches was used to characterize the THz transmission spectra of the materials. Terahertz radiation was generated and detected using photoconductive switches driven by 10 mW optical pulse train from a 780 nm, 100 fs, 75 MHz Ti: Sapphire oscillator ultrafast laser pulse system (Spectra Physics, Owen, CA, USA). The emitted terahertz radiation was collimated by a high-resistivity silicon lens and parabolic mirrors. The samples were measured at ambient temperature in a dry nitrogen atmosphere at 25 °C. The relative humidity of the sample cavity was always kept below 1% by purging high-purity nitrogen gas during spectral measurements, in order to reduce efficiently the effect of strong absorption of atmospheric water vapor. A total of three THz spectra representing three complete sets of sample and reference measurements were averaged for each final spectrum. The time-domain of the THz electric field was recorded for the reference (without sample holder) and each sample, and then after the fast Fourier transform (FFT), the THz spectral absorption was obtained by dividing the sample frequency response by that of the reference. The experimental setup and material parameter extraction have been described in detail in previous work [27].

### 3.3. Theoretical Calculations

Quantum chemistry DFT calculations were performed to simulate the structures of PZA, 3-hBA, and their cocrystal by the Gaussian 09 program [28]. The geometry optimization was carried out with the B3LYP method [29,30,31]. Reliability of the B3LYP functional in calculations of the ground state geometries has been widely assessed previously [32]. The vibrational frequencies of all the molecular systems were calculated based on the 6-311G (d, p) basis set and the calculated wavenumbers were scaled with a factor of 0.96 when using the B3LYP method [33]. As for the THz and Raman simulated spectra, lorentzian line shapes were convolved into the calculated modes using a full-width half-maximum (FWHM) value of 4.0 cm^−1^.

## 4. Conclusions

The cocrystal formation offers an opportunity to modify the key pharmaceutical properties of APIs, therefore it is reasonable to expect that pharmaceutical cocrystal will play an important role in the pharmaceutical industry. In this work, we have recorded the vibrational spectra of PZA, 3-hBA, and their cocrystal using Raman and THz spectroscopic techniques. Raman and THz spectroscopy are complementary to show the vibrational information of the structure of the cocrystal, which can distinguish different molecular conformations and/or configurations. The information reveals that the hydrogen bond formation was formed by the amino group from PZA and the carbonyl group from 3-hBA upon cocrystallization. These vibrational spectra were simulated by DFT calculations, which provided rich information about molecular structures and inter-molecular interactions of the cocrystal between PZA and 3-hBA. The result offers us the unique means and a benchmark to obtain and analyze the molecular structures of cocrystal compounds in pharmaceutical fields.

## Figures and Tables

**Table 1:** Vibrational modes assignment of cocrystal between PZA and 3-hBA.

Mode	Experimental Result/THz	Theoretical Calculation/THz	Vibrational Mode Assignment
a	—	0.40	PZA and 3-hBA molecular out of plane bending vibration
b	0.76	0.81	R1, R2, H12–N11–H13 out of plane bending vibration
c	1.47	1.47	PZA and 3-hBA molecular in plane bending vibration
d	1.66	1.61	PZA and 3-hBA molecular out of plane bending vibration

**Table 2:** Vibrational modes assignment for Raman characteristic peaks of the cocrystal between PZA and 3-hBA.

Mode	Theoretical Wavenumber/cm^−1^	Experimental Wavenumber/cm^−1^	Mode Assignment
ν_1_	237	246	ω (C18–H19, C14–H25, C16–H21, O22–H23)
ν_2_	260	265	Def (R_2_), ρ (H12–N11–H13, C9=O10)
ν_3_	344	-	ρ (O22–H23)
ν_4_	374	386	ρ (H29–O28–C27=O30, H12–N11–H13, C15–O22–H23)
ν_5_	405	405	ρ (C16–H21, C15–O22–H23, H12–N11–H13)
ν_6_	427	431/450	ω (C17–H20, C14–H25, C16–H21)
ν_7_	511	513	ρ (H12–N11–H13, C9=O10, C16–H21, C15–O22–H23)
ν_8_	526	526	Def (R_1_), ρ (12H–N11–H13, C15–O22–H23, C9=O18)
ν_9_	560	552	ω (H12–N11–H13)
ν_10_	625	625	Def (R_2_), ρ (H12–N11–H13, C9=O10)
ν_11_	666	663	Def (R_1_, R_2_), ρ (H12–N11–H13, O30=C27–O28–H29)
ν_12_	770	762	Def R_1_, δ (O30=C27–O28–H29)
ν_13_	819	813	ω (N12–H11–H13)
ν_14_	890	864/908	ω (C14–H25, C16–H21, C4–H7, C5–H8)
ν_15_	940	934	ω (O28–H29)
ν_16_	998	998	Def (R_1_)
ν_17_	1024	1026	Def (R_2_)
ν_18_	1054	1055	Def (R_2_)
ν_19_	1079	1071	ρ (C18–H19, C16–H21, C14–H25, C17–H20, C22–H23)
ν_20_	1119	1096	δ (H12–N11–H13)
ν_21_	1179	1166/1185	δ (H21–C16=C17–H20), ρ (C18–H19, O22–H23, C14–H25)
ν_22_	1293	1300	ρ (O28–H29, C4–H7, C10–H19, C14–H29, C17–H20))
ν_23_	1324	1316	ρ (C14–H25, C17–H20, C18–H19, C16–H21, O22–H23, O28–H29)
ν_24_	1388	1386	ρ (N11–H13, C4–H7, C5–H8, C2–H24)
ν_25_	1433	1433	δ (H13–N11–C9, H29–O28–C27=O30), τ (-NH_2_), ρ (C5–H8)
ν_26_	1453	1450	δ (H13–N11–C9), ρ (O28–H29, C16–H21, C18–H19, C5–H8)
ν_27_	1468	1478	δ (H30=C27–O28–H29), ρ (H13–N11, C16–H21, C4–H7, C5–H8, C17–H20) θ (C26–C27)
ν_28_	1555	1530	Def(R_2_)
ν_29_	1575	1580	δ (H12–N11–H13)
ν_30_	1590	1593	Def (R_2_)
ν_31_	1623	1610	Def (R_1_)
ν_3__2_	1693	1693	θ (C27=O30, C9=H10), δ (H12–N11–H13, C27–O28–H29)

θ-stretching vibration, ρ-in plane bending vibration, ω-out of plane bending vibration, δ-scissoring vibration, Def-deformation, R_1_-the six ring in 3-hBA, R_2_-the six ring in PZA.

**Table 3:** Change of characteristic bond lengths between PZA, 3-hBA, and their cocrystal.

Chemical Bond	Bond Length/Å		
	PZA	3-hBA	Cocrystal
C27=O30	-	1.225	1.258
C27–O30	-	1.431	1.323
O28–H29		0.963	1.000
N11–H13	0.998		1.022
N11–C9	1.467		1.334
C9=O10	1.238		1.259
